# *catena*-Poly[[trimethyl­tin(IV)]-μ-3,5-difluoro­benzoato-κ^2^ *O*:*O*′]

**DOI:** 10.1107/S1600536811025608

**Published:** 2011-07-06

**Authors:** Hong Liu, Handong Yin

**Affiliations:** a College of Chemistry and Chemical Engineering, Liaocheng University, Shandong 252059, People’s Republic of China

## Abstract

In the title compound, [Sn(CH_3_)_3_(C_7_H_3_F_2_O_2_)]_*n*_, the central Sn atom is coordinated by two O atoms from the anion and three methyl C atoms in a polymeric fashion owing to the presence of bidentate bridging carboxyl­ate ligands. The five-coordinate Sn atom exists in a distorted trigonal–bipyramidal geometry with the mol­ecules connected by weak C—H⋯F inter­moleclar inter­actions, forming supra­molecular chains parallel to [010].

## Related literature

For industrial applications and the biological activity of organotin compounds, see: Duboy & Roy (2003 ▶). For related trimethyl carboxyl­ates with similar structures, see: Tiekink, (1994 ▶). 
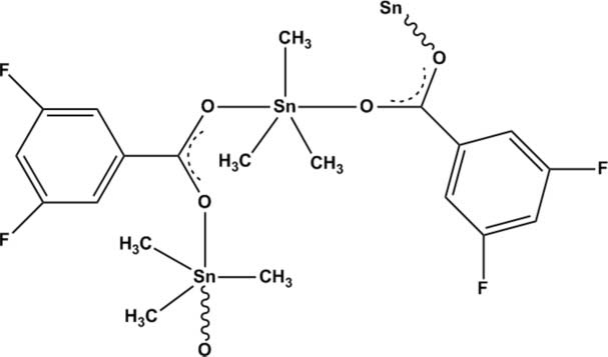


## Experimental

### 

#### Crystal data

[Sn(CH_3_)_3_(C_7_H_3_F_2_O_2_)]
*M* *_r_* = 320.89
Monoclinic, 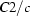

*a* = 13.1371 (12) Å
*b* = 10.0847 (11) Å
*c* = 18.9643 (19) Å
β = 101.864 (1)°
*V* = 2458.8 (4) Å^3^
*Z* = 8
Mo *K*α radiation
μ = 2.08 mm^−1^
*T* = 298 K
0.50 × 0.42 × 0.40 mm

#### Data collection

Siemens SMART CCD area-detector diffractometer
Absorption correction: multi-scan (*SADABS*; Sheldrick, 1996 ▶) *T*_min_ = 0.422, *T*_max_ = 0.490
5945 measured reflections
2165 independent reflections
1725 reflections with *I* > 2σ(*I*)
*R*_int_ = 0.034

#### Refinement

*R*[*F* ^2^ > 2σ(*F* ^2^)] = 0.032
*wR*(*F* ^2^) = 0.073
*S* = 1.17
2165 reflections
140 parameters
H-atom parameters constrained
Δρ_max_ = 0.76 e Å^−3^
Δρ_min_ = −0.69 e Å^−3^

### 

Data collection: *SMART* (Siemens, 1996 ▶); cell refinement: *SAINT* (Siemens, 1996 ▶); data reduction: *SAINT*; program(s) used to solve structure: *SHELXS97* (Sheldrick, 2008 ▶); program(s) used to refine structure: *SHELXL97* (Sheldrick, 2008 ▶); molecular graphics: *SHELXTL* (Sheldrick, 2008 ▶); software used to prepare material for publication: *SHELXTL*.

## Supplementary Material

Crystal structure: contains datablock(s) I, global. DOI: 10.1107/S1600536811025608/jj2092sup1.cif

Structure factors: contains datablock(s) I. DOI: 10.1107/S1600536811025608/jj2092Isup2.hkl

Additional supplementary materials:  crystallographic information; 3D view; checkCIF report

## Figures and Tables

**Table 1:** Hydrogen-bond geometry (Å, °)

*D*—H⋯*A*	*D*—H	H⋯*A*	*D*⋯*A*	*D*—H⋯*A*
C9—H9*C*⋯F1^i^	0.96	2.62	3.470 (7)	148

Symmetry code: (i) 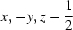.

**Fig. 1.**
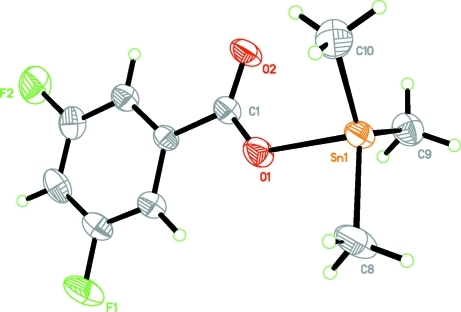
ORTEP diagram of the title compound, with 50% probability displacement ellipsoids.

**Fig. 2.**
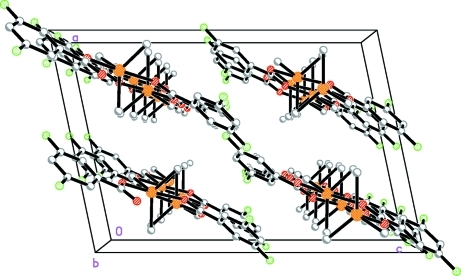
The crystal packing of the title compound, viewed along the b axis. H atoms have been omitted for clarity.

## supplementary crystallographic information

### Comment

In recent years, organotin compounds have attracted increasing attention owing to their wide industrial applications and biological activities (Duboy and Roy, 2003). In continuation of our work in this area, we present the crystal structure of a new compound, (I), C_10_H_12_F_2_O_2_Sn. Similar structures for related trimethyl carboxylates have been reported. (Tiekink, 1994).

In the title compound, (I), the central Sn atom is coordinated by two oxygen atoms from the ligand and three carbon methyl atoms in a polymeric fashion owing to the presence of bidentate bridging carboxylate ligands (Fig. 1). The Sn(1)—O(1) and Sn(1)—O(2)#1 (#1 = -*x* + 1/2,*y* - 1/2,-*z* + 1/2) distances are 2.137 (3)Å and 2.540 (4) Å, respectively. The angle of the axial O(1)—Sn(1)—O(2)#1 is 175.92 (13)°, which deviates slightly from linearity. The five-coordinate Sn atom exists in a distorted trigonal bipyramidal geometry with the molecules connected by weak C—H···F intermoleclar interactions, forming one-dimensional supramolecular chains along the (101) plane (Fig. 2).

### Experimental

3,5-difluorobenzoic acid (0.4 mmol) was added to a methanol solution of sodium ethoxide (0.4 mmol) and heated at reflux for 0.5 h. To this solution was added trimethyltin chloride (0.8 mmol) in benzene and the mixture was refluxed for 8 h, cooled and filtered. The filtrate was evaporated *in vacuo*. The obtained solid was recrystallized from dichloromethane-petroleum ether. Anal. Calcd (%) for C_10_H_12_F_2_O_2_Sn (Mr = 320.89): C, 37.43; H, 3.77; Found (%): C, 38.21; H, 3.92.

### Refinement

The H atoms were positioned geometrically and refined using the riding model with C–H = 0.93 Å, for aromatic H, 0.96 Å for methyl H atoms. The *U*_iso_ parameters for H atoms were constraned to be 1.5*U*_eq_ of the carrier atom for the methyl H atoms and 1.2*U*_eq_ of the carrier atom for the remaining H atoms

### Crystal data

**Table:**

[Sn(CH_3_)_3_(C_7_H_3_F_2_O_2_)]	*F*(000) = 1248
*M**_r_* = 320.89	*D*_x_ = 1.734 Mg m^−^^3^
Monoclinic, *C*2/*c*	Mo *K*α radiation, λ = 0.71073 Å
Hall symbol: -C 2yc	Cell parameters from 3905 reflections
*a* = 13.1371 (12) Å	θ = 2.7–26.3°
*b* = 10.0847 (11) Å	µ = 2.08 mm^−^^1^
*c* = 18.9643 (19) Å	*T* = 298 K
β = 101.864 (1)°	Block, colorless
*V* = 2458.8 (4) Å^3^	0.50 × 0.42 × 0.40 mm
*Z* = 8	

### Data collection

**Table:**

Siemens SMART CCD area-detector diffractometer	2165 independent reflections
Radiation source: fine-focus sealed tube	1725 reflections with *I* > 2σ(*I*)
graphite	*R*_int_ = 0.034
φ and ω scans	θ_max_ = 25.0°, θ_min_ = 2.7°
Absorption correction: multi-scan (*SADABS*; Sheldrick, 1996)	*h* = −14→15
*T*_min_ = 0.422, *T*_max_ = 0.490	*k* = −9→12
5945 measured reflections	*l* = −22→22

### Refinement

**Table:**

Refinement on *F*^2^	Secondary atom site location: difference Fourier map
Least-squares matrix: full	Hydrogen site location: inferred from neighbouring sites
*R*[*F*^2^ > 2σ(*F*^2^)] = 0.032	H-atom parameters constrained
*wR*(*F*^2^) = 0.073	*w* = 1/[σ^2^(*F*_o_^2^) + (0.0148*P*)^2^ + 7.4343*P*] where *P* = (*F*_o_^2^ + 2*F*_c_^2^)/3
*S* = 1.17	(Δ/σ)_max_ = 0.008
2165 reflections	Δρ_max_ = 0.76 e Å^−^^3^
140 parameters	Δρ_min_ = −0.69 e Å^−^^3^
0 restraints	Extinction correction: *SHELXL97* (Sheldrick, 2008), Fc^*^=kFc[1+0.001xFc^2^λ^3^/sin(2θ)]^-1/4^
Primary atom site location: structure-invariant direct methods	Extinction coefficient: 0.00219 (15)

### Fractional atomic coordinates and isotropic or equivalent isotropic displacement parameters (Å^2^)

**Table:**

	*x*	*y*	*z*	*U*_iso_*/*U*_eq_	
Sn1	0.21709 (3)	−0.11988 (3)	0.275296 (18)	0.04976 (17)	
O1	0.1757 (3)	0.0218 (3)	0.34840 (19)	0.0620 (10)	
O2	0.2446 (3)	0.2018 (4)	0.3102 (2)	0.0664 (10)	
F1	−0.0238 (3)	0.1709 (4)	0.5259 (2)	0.0939 (12)	
F2	0.1732 (3)	0.5409 (3)	0.4910 (2)	0.0944 (12)	
C1	0.1945 (4)	0.1461 (5)	0.3495 (3)	0.0483 (12)	
C2	0.1503 (3)	0.2219 (5)	0.4043 (2)	0.0431 (11)	
C3	0.0816 (4)	0.1598 (5)	0.4403 (3)	0.0523 (12)	
H3	0.0613	0.0724	0.4300	0.063*	
C4	0.0443 (4)	0.2301 (6)	0.4913 (3)	0.0587 (14)	
C5	0.0744 (4)	0.3574 (6)	0.5100 (3)	0.0658 (15)	
H5	0.0497	0.4024	0.5459	0.079*	
C6	0.1422 (5)	0.4153 (5)	0.4736 (3)	0.0623 (15)	
C7	0.1796 (4)	0.3514 (5)	0.4209 (3)	0.0539 (13)	
H7	0.2243	0.3947	0.3964	0.065*	
C8	0.1543 (6)	−0.2738 (6)	0.3291 (4)	0.094 (2)	
H8A	0.1335	−0.3461	0.2964	0.141*	
H8B	0.0949	−0.2412	0.3459	0.141*	
H8C	0.2059	−0.3039	0.3693	0.141*	
C9	0.1173 (4)	−0.0367 (6)	0.1847 (3)	0.0685 (16)	
H9A	0.1543	0.0292	0.1635	0.103*	
H9B	0.0587	0.0036	0.1994	0.103*	
H9C	0.0933	−0.1051	0.1501	0.103*	
C10	0.3794 (4)	−0.0930 (6)	0.2996 (3)	0.0766 (18)	
H10A	0.4073	−0.1137	0.2580	0.115*	
H10B	0.4098	−0.1505	0.3386	0.115*	
H10C	0.3951	−0.0025	0.3134	0.115*	

### Atomic displacement parameters (Å^2^)

**Table:**

	*U*^11^	*U*^22^	*U*^33^	*U*^12^	*U*^13^	*U*^23^
Sn1	0.0557 (2)	0.0429 (2)	0.0565 (2)	0.00350 (16)	0.02496 (16)	0.00374 (17)
O1	0.085 (3)	0.044 (2)	0.068 (2)	0.0015 (18)	0.040 (2)	−0.0038 (17)
O2	0.087 (3)	0.059 (2)	0.066 (2)	−0.0043 (19)	0.046 (2)	0.0052 (19)
F1	0.101 (3)	0.099 (3)	0.102 (3)	0.009 (2)	0.070 (2)	0.014 (2)
F2	0.130 (3)	0.058 (2)	0.100 (3)	0.002 (2)	0.033 (2)	−0.0238 (19)
C1	0.055 (3)	0.044 (3)	0.048 (3)	0.007 (2)	0.017 (2)	0.004 (2)
C2	0.042 (3)	0.047 (3)	0.042 (3)	0.007 (2)	0.013 (2)	0.004 (2)
C3	0.056 (3)	0.051 (3)	0.053 (3)	0.004 (2)	0.018 (2)	0.006 (2)
C4	0.058 (3)	0.067 (4)	0.056 (3)	0.013 (3)	0.025 (3)	0.013 (3)
C5	0.077 (4)	0.073 (4)	0.052 (3)	0.031 (3)	0.023 (3)	0.004 (3)
C6	0.075 (4)	0.048 (3)	0.062 (3)	0.016 (3)	0.011 (3)	−0.006 (3)
C7	0.060 (3)	0.051 (3)	0.054 (3)	0.004 (2)	0.018 (2)	0.003 (2)
C8	0.145 (6)	0.049 (3)	0.118 (5)	0.007 (4)	0.097 (5)	0.008 (4)
C9	0.056 (3)	0.083 (4)	0.066 (4)	0.011 (3)	0.013 (3)	−0.002 (3)
C10	0.058 (3)	0.091 (5)	0.078 (4)	0.011 (3)	0.007 (3)	−0.007 (3)

### Geometric parameters (Å, °)

**Table:**

Sn1—C10	2.104 (5)	C4—C5	1.368 (7)
Sn1—C9	2.109 (5)	C5—C6	1.365 (8)
Sn1—C8	2.115 (6)	C5—H5	0.9300
Sn1—O1	2.137 (3)	C6—C7	1.363 (7)
Sn1—O2^i^	2.540 (4)	C7—H7	0.9300
O1—C1	1.278 (6)	C8—H8A	0.9600
O2—C1	1.227 (6)	C8—H8B	0.9600
O2—Sn1^ii^	2.540 (4)	C8—H8C	0.9600
F1—C4	1.353 (6)	C9—H9A	0.9600
F2—C6	1.351 (6)	C9—H9B	0.9600
C1—C2	1.500 (6)	C9—H9C	0.9600
C2—C7	1.379 (6)	C10—H10A	0.9600
C2—C3	1.389 (6)	C10—H10B	0.9600
C3—C4	1.368 (7)	C10—H10C	0.9600
C3—H3	0.9300		
			
C10—Sn1—C9	124.0 (2)	C4—C5—H5	121.5
C10—Sn1—C8	117.8 (3)	F2—C6—C7	119.1 (6)
C9—Sn1—C8	116.5 (3)	F2—C6—C5	118.3 (5)
C10—Sn1—O1	98.80 (19)	C7—C6—C5	122.7 (5)
C9—Sn1—O1	93.67 (19)	C6—C7—C2	119.3 (5)
C8—Sn1—O1	90.07 (19)	C6—C7—H7	120.3
C10—Sn1—O2^i^	84.63 (19)	C2—C7—H7	120.3
C9—Sn1—O2^i^	86.19 (18)	Sn1—C8—H8A	109.5
C8—Sn1—O2^i^	86.33 (19)	Sn1—C8—H8B	109.5
O1—Sn1—O2^i^	175.91 (13)	H8A—C8—H8B	109.5
C1—O1—Sn1	126.3 (3)	Sn1—C8—H8C	109.5
C1—O2—Sn1^ii^	155.9 (3)	H8A—C8—H8C	109.5
O2—C1—O1	124.4 (5)	H8B—C8—H8C	109.5
O2—C1—C2	121.4 (4)	Sn1—C9—H9A	109.5
O1—C1—C2	114.2 (4)	Sn1—C9—H9B	109.5
C7—C2—C3	119.5 (5)	H9A—C9—H9B	109.5
C7—C2—C1	120.8 (4)	Sn1—C9—H9C	109.5
C3—C2—C1	119.7 (4)	H9A—C9—H9C	109.5
C4—C3—C2	118.5 (5)	H9B—C9—H9C	109.5
C4—C3—H3	120.7	Sn1—C10—H10A	109.5
C2—C3—H3	120.7	Sn1—C10—H10B	109.5
F1—C4—C3	118.9 (5)	H10A—C10—H10B	109.5
F1—C4—C5	118.2 (5)	Sn1—C10—H10C	109.5
C3—C4—C5	122.9 (5)	H10A—C10—H10C	109.5
C6—C5—C4	117.0 (5)	H10B—C10—H10C	109.5
C6—C5—H5	121.5		
			
C10—Sn1—O1—C1	59.5 (4)	C1—C2—C3—C4	178.5 (4)
C9—Sn1—O1—C1	−65.8 (4)	C2—C3—C4—F1	179.1 (4)
C8—Sn1—O1—C1	177.7 (4)	C2—C3—C4—C5	−2.2 (8)
Sn1^ii^—O2—C1—O1	133.6 (7)	F1—C4—C5—C6	−179.2 (5)
Sn1^ii^—O2—C1—C2	−46.9 (11)	C3—C4—C5—C6	2.0 (8)
Sn1—O1—C1—O2	−4.0 (7)	C4—C5—C6—F2	180.0 (5)
Sn1—O1—C1—C2	176.5 (3)	C4—C5—C6—C7	−0.1 (8)
O2—C1—C2—C7	−11.4 (7)	F2—C6—C7—C2	178.3 (4)
O1—C1—C2—C7	168.2 (4)	C5—C6—C7—C2	−1.6 (8)
O2—C1—C2—C3	170.6 (5)	C3—C2—C7—C6	1.5 (7)
O1—C1—C2—C3	−9.9 (6)	C1—C2—C7—C6	−176.6 (4)
C7—C2—C3—C4	0.4 (7)		

Symmetry codes: (i) −*x*+1/2, *y*−1/2, −*z*+1/2; (ii) −*x*+1/2, *y*+1/2, −*z*+1/2.

### Hydrogen-bond geometry (Å, °)

**Table:**

*D*—H···*A*	*D*—H	H···*A*	*D*···*A*	*D*—H···*A*
C9—H9C···F1^iii^	0.96	2.62	3.470 (7)	148

Symmetry codes: (iii) *x*, −*y*, *z*−1/2.
